# The Cleveland–Cuyahoga County Food Policy Coalition: “We Have Evolved”

**DOI:** 10.5888/pcd12.140538

**Published:** 2015-06-04

**Authors:** Colleen C. Walsh, Morgan Taggart, Darcy A. Freedman, Erika S. Trapl, Elaine A. Borawski

**Affiliations:** Author Affiliations: Morgan Taggart, Ohio State University Extension, Cuyahoga County, Cleveland, Ohio; Darcy A. Freedman, Erika S. Trapl, Elaine A. Borawski, School of Medicine and Prevention Research Center for Healthy Neighborhoods, Case Western Reserve University, Cleveland, Ohio. When the research was conducted, Dr Walsh was affiliated with the Prevention Research Center for Healthy Neighborhoods, School of Medicine, Case Western Reserve University. Ms Taggart is now with St. Clair Superior Development Corporation, Cleveland, Ohio.

## Abstract

Several pieces of legislation passed in Cleveland, Ohio, from 2007 to 2011, focused on improving the city’s food environment through urban agriculture initiatives. We used qualitative, case study methods, including interviews with 7 key informants, to examine the policy development process and investigate the role of the Cleveland–Cuyahoga County Food Policy Coalition in developing and implementing 4 pieces of legislation. In this article, we focus on 2 pieces of legislation: zoning designation of an urban garden and allowance of small farm animals and bees on residential property. Five key themes emerged: impetus for policy came from community needs; education and raising awareness helped mitigate barriers; a cultural shift took place among policy makers; social connections and individual champions were needed; and concerns over food access and health influenced policy decisions. Legislative actions are important tools to influence the nutrition environment, as long as they are based on local needs and context.

## Food Policy and Health

Consistent availability and affordability of nutritious food is a problem in urban neighborhoods, resulting in systematic injustices related to health outcomes (1). Food policy councils represent one strategy for creating policy, systems, and environmental changes to promote health by enhancing access to nutritious foods (2). The Cleveland–Cuyahoga County Food Policy Coalition (CCCFPC), founded in 2007, has been noted for its role in policy gains (3–6).

Food policy refers to a broad set of actions or decisions by government bodies, businesses, or organizations that have an impact on the production, distribution, and consumption of food (5). In this article, food policy refers to actions taken by local government in the form of legislation implemented in Cleveland to improve the city’s food environment through urban agriculture ordinances. The objective of this case study was to describe the successes and challenges of creating the policies and the role of CCCFPC in the policy-making process.

## Key Informant Interviews

We used qualitative, case study methods to explore food-related policies adopted by Cleveland from 2007 to 2011. Data related to each policy (ie, evaluation reports of the CCCFPC, the ordinances as posted in the City Record of Cleveland) were collected, and semistructured interviews were conducted with key informants. To identify policies, we compiled a list of CCCFPC initiatives from the previous 6 years. At the time of data collection (February–June 2013), CCCFPC had been involved in 20 food policy initiatives. These policies ranged from informal recommendations and guidelines for organizations, businesses, and governments, to formal legislative actions (6). Of the 20 policies reviewed, 6 resulted in legislation adopted by the City of Cleveland, a criterion we used to select 4 cases for this study (Box).

**Box. Selected Food Policy Cases in Cleveland, Ohio, 2007–2011**

**Table:** 

Case Name (Year Passed)	Description of Policy	Organizations Represented by Interviews With Key Informants
Urban Garden District Zoning (2007)	Makes it possible for a parcel of land to be designated as a community garden. Rezoning a garden, however, does not guarantee that it can never be lost. It simply makes replacing a garden a public process, giving neighbors a voice to protect it.	The Ohio State University Extension, Cuyahoga County; Cleveland Botanical Garden; Cuyahoga Community Land Trust; Cleveland City Council; Cleveland City Planning
Keeping of Farm Animals and Bees Licensing and Restrictions (“Chickens and Bees”) (2009)	Allows for the keeping of small farm animals (goats, pigs, sheep, ducks, chickens, rabbits and similar animals) and bees on residential property in Cleveland.	The Ohio State University Extension, Cuyahoga County; Cleveland City Council; Cleveland City Planning
Agriculture and Farm Stands in Residential Districts (2010)	Agriculture as principal use on all vacant residentially zoned lots. Also permits the sale of produce from farm stands in Residential Districts.	The Ohio State University Extension, Cuyahoga County; Cleveland City Council; Cleveland City Planning
Mobile Food Vending (“Food Truck Legislation”) (2011)	Allows mobile food trucks to operate within city limits.	Cleveland City Planning

The CCCFPC organizer (M.T.) made initial suggestions that helped to identify 10 key informants representing local government and community organizations associated with each policy and the CCCFPC. Seven of the 10 key informants agreed to participate and provided informed consent; 3 declined participation because of their perceived lack of insights into the legislation or because they did not have supervisory permission to participate. We limited our analysis to 4 pieces of legislation to focus on those that involved the key informants and that represented policies with distinct food-related objectives (Box). To demonstrate themes emerging from the case study, we focused this article on 2 policies: Urban Garden District Zoning and Keeping of Farm Animals and Bees Licensing and Restrictions (henceforth “Chickens and Bees”).

During the interviews, which lasted approximately 1 hour, we explored the following topics: the impetus for the policy action; how legislation was created and implemented; the people involved; the perceived role of the CCCFPC during the process; if and how results of the legislation were being tracked; how participants saw this policy as improving urban health; and how legislation fit within broader city or regional goals. One researcher (C.C.W.) conducted all interviews in person except one, which was conducted by telephone because the participant no longer lived in Cleveland. The 7 participants represented 5 organizations: The Ohio State University Extension, Cuyahoga County; Cleveland Botanical Garden; Cuyahoga Community Land Trust; Cleveland City Council; and Cleveland City Planning. Participants included junior- and mid-level employees as well as high-ranking members of city government. Three participants were knowledgeable about and provided input on all 4 policies; 4 participants were interviewed primarily about 1 piece of legislation.

Interviews were audiorecorded, professionally transcribed, and reviewed for accuracy. Using a narrative analysis approach, we analyzed the qualitative data inductively to identify themes in participant narratives related to understanding common successes and challenges and the role of CCCFPC in the legislative process. One coder (C.C.W.) primarily conducted data analysis, and the codes were reviewed with a research assistant to ensure credibility and confirm key themes (7). Case Western Reserve University’s institutional review board approved this research.

## Key Themes

We found 5 underlying themes related to the successful passage of all 4 policies and CCCFPC’s role.

**Impetus for each policy came from the community or the needs of residents, and the CCCFPC played a role in making these needs heard.** The 4 policies provided solutions to legislative obstacles for residents. For the Urban Garden District Zoning legislation, 4 study participants representing 3 community organizations explained how they approached a council member about the need to create a garden preservation strategy after they had seen several long-time gardens, viewed as vital to their communities, razed for development. The participants recalled a particular instance when a developer sought to demolish a garden that had been a part of the neighborhood since before World War II to make space for a parking lot.

Having seen this same scenario unfold in other neighborhoods, these 4 participants and the councilman decided to pursue a garden preservation strategy, which eventually became the Urban Garden District Zoning policy. Participants explained that although the zoning policy does not offer full protection from development, it does necessitate a public hearing process should someone try to change the zoning category to allow for any other use of the property. Informants indicated that initiators of this policy had pushed for more binding and legal land preservation for gardens, “something with more teeth,” one participant said. However, because of perceived political barriers, the creation of the zoning district was deemed most feasible.

Interviewees indicated that the Chickens and Bees legislation emerged as momentum on urban agriculture was building locally and nationally. Participants recalled 2 gardeners who wanted to raise chickens for eggs to sell as part of their Community Supported Agriculture program but who repeatedly received citations (for health code violations) from the city. These gardeners worked with the CCCFPC, using their stories as a way to convince policy makers of the community and economic development potential of allowing such endeavors. One participant described these community voices, which were heard by the city council and the planning commission at public hearings, as a catalyst to begin to examine the issues related to the use of policy for urban agriculture to find a balance between the needs and interests of residents, those of public health officials, and those of the community food systems advocates who wanted more food production in Cleveland.

**Education and raising awareness helped mitigate barriers.** Participants described the need to educate policy makers to achieve success and the importance of CCCFPC in raising awareness. Education was discussed as particularly important for passing the Chickens and Bees policy because officials raised concerns over health and safety. According to participants, opponents voiced concern about disease, hygiene, and the vulnerability of residents with bee allergies. The eventual passage was perceived to depend on the presentation by university-based experts on bee behavior to quell fears and provide information needed to build safety precautions into the legislation. These protections, which became part of the ordinance, include the need for larger setbacks for bigger animals (eg, pigs, sheep), guidelines for placement of coops and cages, and the need for a water source and flyaway barriers for bees. Urban farmers wanting to raise livestock or bees are required to obtain a license from the Cleveland Department of Public Health, a protection that interviewees indicated was important for passage.

Participants also described education as important in the process of developing and passing the Urban Garden District Zoning:

I think that it was educating people about who gardeners really were . . . how many places in the city there were community gardens, and the value that they had to people, frankly, to people of limited income . . . some of it was access to fresh food, but a lot of it was the economic issue for a family.

**Social networks and social capital of CCCFPC members were crucial, and political will and champions were needed. **The working relationships between CCCFPC members exemplified the importance of social networks in accomplishing policy change. Individuals from partner organizations, who worked directly with diverse, often marginalized communities, described how they leveraged their personal and professional relationships to build trust between residents and policy makers. Participants indicated that a significant amount of time was spent in formative meetings developing these relationships, because they were seen as integral to accomplishing policy that would meet the most needs. An inclusive, open-membership grassroots approach allowed for a more “organic” creation of the CCCFPC with people, “not just the ‘suits,’” and contributed to its success. (In other regions, food policy councils are formal councils created by government bodies in which members are appointed or invited [8]). Those interviewed suggested that the informal style and intentional process of formation of the CCCFPC might have played a role in early policy gains.

Participants described the importance of individual political champions willing to push urban agriculture initiatives. Charismatic leaders in both the CCCFPC and city government were willing to take risks to pursue each policy. According to interviewees, although many public officials today are quick to lend support to popular urban agriculture initiatives, at the time these 2 ordinances passed, there were vocal opponents on city council who worried about what this would mean for perceptions of Cleveland. One city councilman explained how other council members were worried about “ruralizing the urban core”:

[A] lot of derogatory comments got made by colleagues of mine that were whistling ‘Green Acres’ during the legislation process . . . and you’re like, okay I get it, but you know, I’m sick of being on the front page of the Wall Street Journal for the foreclosure crisis.

The city planning official interviewed was also touted by other participants as having demonstrated strong leadership because the Urban Garden District Zoning legislation was risky for urban planners who typically do not consider “spot zoning” (ie, adjacent parcels with different zoning categories) as good planning practice. A city planning commission official described this policy as an unusual way to offer protection from redevelopment, but the idea of using zoning policy to promote urban agriculture in the city was appealing to him. Participants also described the CCCFPC organizer (M.T.) as key to these policy successes, lauding her for her community organizing and leadership capacities.

**A cultural change and an evolution in attitude about urban agriculture took place in the city. **Participants described an overall culture shift in Cleveland that made these 4 policies possible. The Urban Garden District Zoning legislation was described by all informants as an important success and the beginning of a shift in the way local policy makers viewed urban agriculture. As one city planning official explained, “urban gardens were considered just filling a gap in until the ‘highest and best use’ comes along, and I think now we realize that in many cases, an urban garden or an urban farm is the highest and best use. . . . [W]e have evolved.”

Participants described the role that the process of developing and passing the urban agriculture legislation played in changing not only public officials’ perceptions of community gardens and urban farms but also among businesses and residents in general. As one participant stated, “I think community spaces and gardens are probably much more highly valued within the city now . . . and I think a lot of people would argue it is the best use for large plots of vacant land.” This new attitude was contrasted with the opinions voiced by council members who referred to community gardens as “eyesores” when the Urban Garden Zoning policy was first introduced or those who thought the livestock legislation would decrease property values.

**Food access inequities in marginalized Cleveland neighborhoods were described as important reasons for the local food policies and the work of CCCFPC.** Participants discussed policies in terms of food access and health, and even before being asked about this topic, they described the persistence of food environment inequities, high obesity rates, and health disparities in Cleveland neighborhoods as justification for these policies. The city planning official explained that what he finds most intriguing about these policies is identifying the role that planning can play in addressing lack of access to nutritious food in inner city, low-income neighborhoods.

The council member interviewed for this study was vocal about the urban agriculture movement in Cleveland as being about food justice. When asked about who stands to benefit from these policies, he quoted the Bible: “‘Whatsoever you do for the least of these, you’re doing for me.’ It’ll help the people who are broke, broken, who are in food deserts, who are obese, who are without insurance, who are without access to medical care, the people who people usually forget.” Other participants also described food justice as motivation for their efforts and the vision of the CCCFPC emphasizes the importance of food security for all residents (9).

## Discussion

The results of our interviews suggest that achieving food policy change requires strong leaders, relationship building, and the ability to raise awareness among policy makers and the public. The success of the CCCFPC may, in part, be attributed to its grassroots formation and structure. Although notable exceptions exist (8,10,11), government-appointed food policy councils face bureaucratic challenges and can be less resilient (12). Although food policy councils within government entities may have more access to funding and be able to exert political authority, “their policy recommendations may not be as responsive to community needs” (8). In a case study of the Community Health Councils model in South Los Angeles, Lewis and colleagues (13) concluded that 2 food policy innovations, which sought to address the social determinants of chronic disease, were successfully moved through the policy process because the model used a bottom-up approach to develop community-based strategies and a multisector coalition.

Although our methods created a potential for limited perspectives, our findings reflect input from key players in urban food policy development in Cleveland. Their stories aligned, and we were able to find common threads in their accounts. The policies reviewed in this study, which were some of the first of their kind in the country (14,15), helped foster a local environment in which policy development to enhance access to nutritious foods flourished. As participants explained, the use of policy to promote urban agriculture provided a way to turn negative headlines about foreclosures and vacant land into something positive and innovative. The CCCFPC played a central role in these policy changes by collecting information, developing policy recommendations, and raising awareness to shape the local food environment.

Our findings indicate that legislative actions are important tools to influence the food environment, as long as they are based on local needs and context. Part of the success of these policies is due to their being grounded in the sociodemographic and political realities of Cleveland. Most community gardeners in Cleveland are older, low income, and African American (16,17). In some cities, such as Seattle, property is at such a high demand that the cost of a garden plot might be unattainable to low-income residents (18). Because of deindustrialization and population declines over the last century and the foreclosure and economic crisis of the last decade, vacant land on which gardens can thrive at little or no cost to residents is abundant in Cleveland (19,20). The special sociodemographic and economic characteristics of Cleveland have shaped the urban agricultural landscape and influenced the types of policy changes that the CCCFPC was able to seek and accomplish. As food policy councils continue to be promoted as a means to improve nutritious food access, policy identification should be highly contextualized and reflect of the needs of communities they seek to serve.

Whether or not these policies have increased access to fresh and nutritious food in Cleveland to overcome inequities in the system, the participants viewed that goal as an important reason for creating such policies and for the work of the CCCFPC. Future research focused on assessing the impact of policy efforts on nutritious food access and health outcomes is needed.
